# Increased Targeting Area in Tumors by Dual-Ligand Modification of Liposomes with RGD and TAT Peptides

**DOI:** 10.3390/pharmaceutics14020458

**Published:** 2022-02-21

**Authors:** Mohamadreza Amin, Mercedeh Mansourian, Peter C. Burgers, Bahareh Amin, Mahmoud Reza Jaafari, Timo L. M. ten Hagen

**Affiliations:** 1 Laboratory Experimental Oncology, Department of Pathology, Erasmus University Medical Center, 3015 GD Rotterdam, The Netherlands; t.l.m.tenhagen@erasmusmc.nl; 2 Nanomedicine Innovation Center Erasmus (NICE), Erasmus University Medical Center, 3015 GD Rotterdam, The Netherlands; 3 Nanotechnology Research Center, Pharmaceutical Technology Institute, Mashhad University of Medical Sciences, Mashhad 9196773117, Iran; mercedeh.mansourian@intravacc.nl (M.M.); jafarimr@mums.ac.ir (M.R.J.); 4 Department of Pharmaceutical Nanotechnology, School of Pharmacy, Mashhad University of Medical Sciences, Mashhad 9177948954, Iran; 5 Laboratory of Neuro-Oncology, Department of Neurology, Erasmus MC University Medical Center Rotterdam, 3015 CN Rotterdam, The Netherlands; p.burgers@erasmusmc.nl; 6 Cellular and Molecular Research Center, Department of Physiology and Pharmacology, Faculty of Medicine, Sabzevar University of Medical Sciences, Sabzevar 9613873136, Iran; aminb@medsab.ac.ir

**Keywords:** PEGylated liposomes, tumor cell targeting, vascular targeting, active targeting, dual-targeted nanoparticle, TAT peptide, RGD peptide, tumor heterogeneity

## Abstract

Modification with polyethylene glycol (PEGylation) and the use of rigid phospholipids drastically improve the pharmacokinetics of chemotherapeutics and result in more manageable or reduced side-effects. A major drawback is retarded cellular delivery of content, which, along with tumor heterogeneity, are the two main obstacles against tumor targeting. To enhance cellular delivery and reach a bigger area of a tumor, we designed liposomes decorated with two ligands: one for targeting tumor vasculature via a cyclic-pentapeptide containing arginine-glycine-aspartic acid (RGD), which impacts tumor independent of passive accumulation inside tumors, and one for extravascular targeting of tumor cells via a cell-penetrating peptide derived from human immunodeficiency virus type 1 transactivator of transcription (TAT). Liposomes with different ligand combinations were prepared and compared with respect to performance in targeting. Intravital imaging illustrates the heterogeneous behavior of RGD-liposomes in both intravascular and extravascular distribution, whereas TAT-liposomes exhibit a predictable extravascular localization but no intravascular targeting. Dual-ligand modification results in enhanced vascular targeting and a predictable extravascular behavior that improves the therapeutic efficacy of doxorubicin-loaded liposomes but also an augmented clearance rate of liposomes. However, the dual-modified liposome could be a great candidate for targeted delivery of non-toxic payloads or contrast agents for therapeutic or diagnostic purposes. Here we show that the combination of vascular-specific and tumor cell-specific ligands in a liposomal system is beneficial in bypassing the heterogeneous expression of tumor-specific markers.

## 1. Introduction

Liposomes are spherical bilayers resulting from the self-assembly of phospholipids in an aqueous environment. Their application for drug delivery was first proposed by Gregoriadis about half a century ago [1]. Since then, lipid-based nanoparticles for drug delivery have advanced rapidly, which has resulted in a variety of liposomal formulations of chemotherapeutics. A successful example is the first FDA-approved PEGylated liposomal doxorubicin (PLD), which reached the market in 1995 [2]. Doxil was designed with the promise of long circulation in blood and capability of targeting tumors via passive extravasation through the leaky tumor vasculature and retention inside the tumor interstitium because of impaired lymphatic drainage of the tumor, a process which is known as the enhanced permeation and retention (EPR) effect [3]. High stability due to a rigid and impermeable lipid bilayer, stealth behavior because of a hydrophilic PEG coat, a size around 90 nm, highly efficient encapsulation yield, and a high drug-to-lipid ratio due to remote loading of doxorubicin (DXR) are beneficial features of PLD that guarantee long and stable circulation with restricted distribution volume to the blood pool [4], a reduction in lethal side effects [2,5,6,7] of DXR, and increased DXR delivery to tumors compared to administration of free DXR [8,9]. However, improved stealth and stability trade off intracellular drug delivery due to the steric hindrance effect of PEG moieties that minimize liposome–cell interactions and the limited drug release through the rigid liposomal bilayer where drug delivery into cells mainly hinges on accidental uptake or slow drug release from these stable liposomes, which challenges the anti-tumor efficacy of PEGylated liposomes compared to their non-PEGylated counterparts [10,11].

One solution for increasing cellular delivery of PEGylated liposomes is decoration of the liposome surface with ligands that interact with distinct receptors overexpressed in tumor cells upon which liposomes are being taken up and internalized into the cell. This has been coined as “active targeting.” Using the term “active targeting” may mislead some researchers towards smart drug delivery systems that actively accumulate inside tumors and interact with malignant tumor cells. However, it is worth mentioning that active targeting of tumor cells still relies on passive extravasation and accumulation of nanoparticles in the tumor interstitial space where ligand-modified nanoparticles can interact with the targeted receptor. It is important that there is a substantial debate about the usefulness of EPR for enhancing drug delivery to tumors by nanocarriers. The heterogeneity of tumor histology is a major problem for efficient drug delivery, which is not only observed between tumors [12] but also within a tumor, depending on dimension, location, and stage of development [13,14]. In fact, it is now clear that in the clinic, patients mainly benefit from the reduced side effects of nanocarrier-encapsulated drugs due to the alteration of drug biodistribution and not necessarily from improved anti-tumor activity [15,16]. Therefore, tumor targeting of ligand-modified nanoparticles is negatively impacted by the non-predictive and heterogeneous nature of the EPR effect. This, in part, contributes to the clinical failure of many clinically investigated nanomedicines, most particularly ligand-modified nanoparticles. Challenges associated with EPR-based drug delivery have prompted researchers to exploit other modalities that bypass EPR for drug delivery or approaches that enhance EPR at the tumor site [17,18,19,20,21].

One alternative approach to attack tumors that can bypass EPR is anti-vascular targeting, which, in principle, is not dependent on extravasation and the EPR-based accumulation of nanodrugs inside tumors. Anti-vascular targeting aims at the destruction of existing tumor vasculature to deprive tumor cells of a nutrient supply; therefore, destruction of existing tumor vasculature can indirectly affect a large number of tumor cells [22] and is expected to cover a broad spectrum of cancers [23,24,25]. Since tumor neovasculature is accessible to systemic treatment, extravasation into the tumor interstitium is not a prerequisite step, making this approach independent from EPR. In addition, compared to tumor cells, endothelial cells are non-malignant and are relatively genetically stable and therefore are less prone to developing drug resistance [23] or downregulating the expression of existing targeted receptors. However, the heterogeneous expression of vascular markers, such as integrins, that are frequently been used for anti-vascular targeting [26] or anti-angiogenesis [27,28,29] also depend on tumor kind and stage and the location of vessels in the tumor [30], which impose heterogeneity to vascular targeting.

Heterogeneity associated with both tumor cell targeting and tumor vascular targeting prompted us to explore a dual functional liposomal delivery system with the capability of invading tumors via targeting both tumor vessels while circulating in blood and tumor cells after extravasation and accumulation inside the tumor interstitium.

For vascular targeting, a cyclic RGD pentapeptide with a greater performance compared to a series of other RGD pentapeptide composites was selected to direct liposomes towards α_v_β_3_ and α_v_β_5_ integrins [31,32], which are crucially involved in the angiogenesis of solid tumors and are overexpressed in tumor neovasculature during angiogenesis [28,33].

In order to enhance the internalization of liposomes into tumor cells, an analog of a transactivator of the transcription of an HIV-1 (TAT) peptide was used. TAT belongs to the family of cell-penetrating peptides (CPP) and has been shown to enhance the cellular uptake of nanomedicines [34,35,36,37] via receptor-mediated endocytosis [38].

To this end, PEGylated liposomal doxorubicin (PLD) or fluorescently labeled-PEGylated liposomes (FPL) were decorated with different densities (ligands per liposome) of either an RGD peptide, a TAT peptide, or both. The capability of different peptide-modified preparations to interact with a variety of cancer cells and human umbilical vein endothelial cells was first assessed in vitro by flow cytometry and confocal imaging in living cells. Through intravital confocal microscopy, intratumoral behavior of different ligand-modified preparations were visualized and compared against each other in living animals bearing B16 tumors. Therapeutic efficacy and the biodistribution of different peptide-modified PLD were also assessed and compared against non-modified plain-PLD in the C57 mice model of the B16 tumor.

## 2. Materials and Methods

### 2.1. Materials

Hydrogenated soya phosphatidylcholine (HSPC) and Methoxypolyethyleneglycol (Mw 2000)-distearylphosphatidylethanolamine (mPEG2000-DSPE) were purchased from Lipoid (Ludwigshafen, Germany). Maleimide-PEG2000 distearoylphosphatidylethanolamine (Mal-PEG2000-DSPE) and 1,2-dioleoyl-sn-glycero-3-phosphoethanolamine-N-(lissamine rhodamine B sulfonyl) (Liss Rhod, PE) were purchased from Avanti Polar Lipids (Alabaster, AL, USA). Then, 3,3′-Dioctadecyloxacarbocyanine perchlorate (DiO); 1,1′-Dioctadecyl-3,3,3′,3′-Tetramethylindodicarbocyanine; 4-Chlorobenzenesulfonate Salt (DiD); and LysoTracker Red were purchased from Thermo Fisher Scientific (Waltham, MA, USA). Ammonium sulfate, Cholesterol, and doxorubicin hydrochloride (Dox) were purchased from Sigma-Aldrich (St Louis, MO, USA). RGDf[N-met]C was synthesized by Ansynth Service B.V. (Roosendaal, The Netherlands). A TAT peptide analog equipped with 3G as a spacer and a C for conjugation (CGGG-RKKRRQRRRGYG) was synthesized by Peptron Inc. (Daejeon, South Korea). All other solvents and reagents were used as chemical grade.

### 2.2. Conjugation of Peptides to DSPE–PEG–Maleimide

Thiol-reactive TAT or RGD peptides were first dissolved in DMSO in a concentration of 10 mg/mL and added to a chloroform solution of DSPE–PEG2000–Maleamide. The volumes were adjusted to reach a chloroform:DMSO ratio of 50:50 (*v/v*) with a peptide:lipid molar ratio of 1.1:1.0. The mixture was then incubated overnight at room temperature with gentle stirring under an argon atmosphere. A sample of lipid, peptide, and the reaction mixture was withdrawn and analyzed by MALDI-TOF to confirm the conjugation and consumption of lipid molecules and was finally quantified for phospholipid content using a Bartlett phosphorus assay. Aliquots of a defined amount of lipopeptide in the conjugated mixture were dried by nitrogen gas blowing, followed by freeze drying, sealed under vacuumed condition and stored at −20 °C until use.

### 2.3. MALDI-TOF Measurements

**Figure 1:**
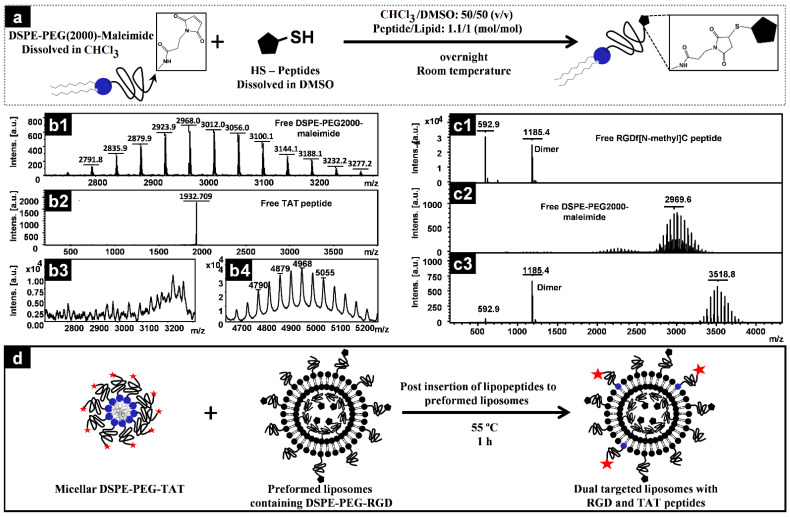
Schematic representation of the coupling reaction of HS-peptides to maleimide-functionalized phospholipids (**a**), the corresponding mass spectral (MALDI-TOF) data for free DSPE-PEG2000-maleimide (**b1**,**c2**), free TAT (**b2**), free RGD (**c1**), consumption of free lipids in reaction mixtures (**b3**,**c3**), and formation of lipopeptide conjugates (**b4**,**c3**). (**d**) illustrates the schematic of preparation of dual-targeted liposomes by postinsertion of TAT-lipopeptide into preformed RGD-liposomes.

MALDI-TOF spectra were recorded on an Ultraflex III MS (Bruker Daltonics) mass spectrometer operated in the positive ion mode using the reflection mode (mass range up to *m*/*z* 4000) or linear mode (masses above *m*/*z* 4000, Figure 1(b4)) and DHB (2,5-dihydroxybenzoic acid; 10 mg/mL in water) as the matrix [39].

### 2.4. Preparation and Characterization of Liposomes

Liposomes were prepared by the solvent evaporation, plus sonication and extrusion, and DXR was encapsulated in liposomes by the remote loading method using the ammonium sulfate gradient technique described elsewhere [40]. Briefly, a HSPC/Cholesterol/m-PEG-DSPE/ lipophilic dye (56.1:38.2:5.5:0.2 mol%) was first dissolved in chloroform; dried by a rotary evaporator and overnight connection to a freeze dryer; and hydrated by ammonium sulfate 250 mM for DXR remote-loading or a HEPES 10 mM, NaCl 140 mM, and pH 7.4 buffer for DXR-free preparations (FPLs). The liposomal suspensions were then bath-sonicated for 5 min at 45 KHz and passed through polycarbonate membranes of 200 and 80 nm sequentially, using LIPEXTM (Northern Lipid Inc. Vancouver, Canada), all at 65 °C. Liposomes encapsulating ammonium sulfate were first dialyzed against sucrose 10% to create the ammonium gradient, then incubated with DXR solution (1 mg DXR per 10 µmol of total lipid) at 65 °C for 60 min, cooled to room temperature, and dialyzed against sucrose 10% to remove free DXR. The loading efficiency of DXR into liposomes was calculated based on dividing the DXR:lipid ratio after the separation of free drug to DXR:lipid ratio before the separation of free DXR.

For RGD-containing preparations the required amount of RGD-PEG-DSPE (600 peptides/liposome or 300 peptides on the surface of liposome) was added to the initial chloroform mixture while the amount of the added RGD-PEG-DSPE was subtracted from the mPEG-DSPE content of the preparation. TAT modifications were made by postinserting the required amount of TAT-PEG-DSPE into preformed liposomes by incubation of the micellar TAT-lipopeptide, dispersed in distilled water with preformed liposomes for 1 h at 55 °C (schematically depicted in Figure 1). Estimations of the required amount of lipopeptides were made based on liposomes of 100 nm composed of 80,000 phospholipid molecules. The preparations were finally dialyzed against sucrose 10% for PLDs or HEPES/NaCl for FPL to remove free DXR or the free peptides that were added excessively during conjugation. For details of each liposomal preparation, see Table 1.

### 2.5. Leakage Stability Assessments

The leakage stability of PLDs was evaluated over 48 h of incubation at 37 °C in presence of 30% fetal calf serum (FCS). Briefly, 500 µL of liposomes were diluted in 3 mL of sucrose 10% and 1.5 mL of FCS; transferred to a Slide-A-Lyzer dialysis cassette with a 3.5 kD cut-off size (Pierce, Rockford, IL, USA); and incubated at 37 °C inside a sterile-sealed beaker filled with 100 mL of sucrose 10%, FCS 30%, and NaN_3_ 2% with continuous stirring. Samples of dialysis medium were withdrawn at different time points, and the amount of released DXR was assayed spectrofluorometerically. The percentage of DXR remaining encapsulated was calculated.

### 2.6. Cell Culturing

Human umbilical vein endothelial cells (HUVEC) were isolated by collagenase digestion using the method described by Jaffe [41] and were cultured in RPMI 1640 (Sigma-Aldrich Co., London, UK) containing 20% (*v/v*) FCS. C26 murine colon carcinoma cells (Cell Line Services GmbH, Eppelheim, Germany) were cultured in RPMI 1640 containing 10% (*v/v*) FCS. B16F0 murine melanoma (Sigma-Aldrich Co., Gillingham, UK), human basal epithelial MDA-MB-231 and luminal-epithelial MCF-7 breast carcinoma cells [42] (both were kindly provided by Dr. John W.M. Martens, Erasmus Medical Center, Rotterdam, The Netherlands) and human melanoma BLM cells [43] were cultured in Dulbecco′s Modified Eagle′s Medium (DMEM) (Sigma-Aldrich Co., London, UK) containing 10% FCS. All cell lines were cultured at 37 °C in a 5% CO_2_/95% air humidified atmosphere, and all the mediums were supplemented with 25 mM of HEPES, 2 mM of l-glutamine, 100 IU/mL of penicillin, and 100 mg/mL of streptomycin.

### 2.7. In Vitro Cellular Association of Peptide-Modified Liposomes

To analyze the capability of RGD and TAT peptides in enhancing the cellular delivery of liposomes, cells including C26 colon carcinoma, B16F0 murine melanoma, BLM human melanoma, MCF-7, and MDA-MB-231 human breast cancer cells and human umbilical vein endothelium cells (HUVEC) were seeded in a density of 10^5^ cell/well in triplicates in 24-well plates and incubated with DiO-labeled liposomal preparations (100 nmol liposomal phospholipid/ 0.5mL) for 3 h at 37 °C. Cells were then detached by trypsinization, replicates were pooled and washed three times with phosphate buffer saline (PBS) pH 7.4 containing 1% FCS, and volume was adjusted to 500 µL and incubated with 5 µL of propidium iodide (PI) (100 µg/mL) for 15 min at room temperature. Ten thousand cells gated on live cells by FSC/SSC and PI exclusion and assayed for the intensity of green DiO fluorescent using FACS Calibur (Becton-Dickinson, San Jose, CA, USA).

### 2.8. Intravital Imaging

#### 2.8.1. Tumor Model

In compliance with the protocol approved by the committee on Animal Research of the Erasmus MC, Rotterdam, the Netherlands, 10 million B16 cells were injected subcutaneously in the flanks of C57Bl6 mice, and the mice were housed at 20–22 °C, with a humidity of 50–60%. Bulk tumors of 10 mm in diameter were used for transplantation into C57Bl6 mice, expressing an eNOS-tag-GFP fusion protein constitutively in their vascular endothelium. Tumor pieces were implanted in a dorsal skin flap window chamber for intravital imaging [44,45]. Window chamber-bearing mice were used for experiments after 8–12 days of tumor implantation when tumor size reached 4–6 mm in diameter. These mice were housed in an incubator room with a humidity of 70% and a temperature of 30–32 °C.

#### 2.8.2. In Vivo Imaging

In vivo behavior of fluorescently labeled peptide-modified PEGylated liposomes (FPL) in the tumor region was observed by intravital confocal microscopy on dorsal skin-fold window chamber-bearing mice after an IV injection through the penile or tail vein at a dose of 5 μmol of lipids. The mice were then anesthetized with isoflurane (Nicholas Piramal, London, UK) and placed on a heated stage (37 °C) under the confocal microscope (Zeiss LSM 510 META).

### 2.9. In Vivo Biodistribution

Biodistribution of liposomes was evaluated in C57 mice bearing a B16F0 tumor after a single IV dose of 15 mg/kg of liposomal DXR. Briefly, 15 days after B16F0 tumor inoculation, when the tumors were approximately 7 mm wide, the mice (eight per group) were injected via the tail vein with 15 mg/kg of encapsulated in different PLDs. At 6 and 12 h after the injection, the mice (four at each time point) were deeply anesthetized and sacrificed after blood collection via heart puncture, and tumors, spleens, and parts of livers were dissected. Bloods and organs were treated the same as mentioned elsewhere to extract and assay their DXR concentration [32]. In line and consistent with our previous studies on BALB/c mice [31,38] at time points of 6 h and 12 h after injection, which were found more informative than later time points, were selected to evaluate the biodistribution of different liposomal preparations.

### 2.10. Therapeutic Efficacy

These animal experiments were performed in compliance with the Institutional Ethical Committee and Research Advisory Committee of Mashhad University of Medical Sciences guidelines. On day 0, a subcutaneous tumor was initiated by inoculating 3 × 10^5^ B16 melanoma cells in the right flank of female C57 mice. On day 7 post-inoculation, mice with a palpable tumor received a single tail vein injection of either sucrose 10% solution as a negative control or 15 mg/kg of DXR encapsulated in PLDs. The tumor volume was estimated by measuring the three orthogonal diameters of tumors using the (a × b × c)/2 formula. Mice were monitored for up to 70 days post-inoculation or until one of the following conditions for euthanasia was met: (1) their body weight dropped below 20% of their initial mass, (2) their tumor was greater than 2.0 cm across in any dimension or the tumor volume was greater than 1 cm^3^, (3) they became lethargic or sick and unable to feed, or (4) they were found dead [46,47].

### 2.11. Statistical Analysis

Statistical analyses were performed using GraphPad Prism version 5 (GraphPad Software, San Diego, CA, USA). Survival data were analyzed by the log-rank test. For other comparisons, one-way ANOVA and Newman–Keuls multiple comparisons tests were employed.

## 3. Results

### 3.1. Conjugation of Peptides to PEG-Lipids

Peptides were conjugated to DSPE-PEG-Maleimide based on the reaction depicted in Figure 1a. The free functionalized PEG lipids were almost completely consumed by the reaction with free peptides (Figure 1(b3,c3)), and the corresponding mass of free lipids increased proportionally with the molecular weight of free peptides (Figure 1(b4,c3)), indicating the formation of lipopeptides with an efficiency of about 100% with respect to lipid consumption. Using an excess of HS-peptide guarantees the occupation of all pure maleimide-functionalized lipids. Therefore, the lipopeptide could be simply quantified with a phosphorus assay.

### 3.2. Liposome Characterization and Stability Assessments

The particle size of all PLDs was around 100 nm. While the incorporation of peptides caused no impact on the liposomes size, the presence of the cationic TAT peptide on the surface of the liposomes slightly reduced the negative ζ-potential of liposomes (Table 1). The loading efficiency of DXR into all liposomes was identically around 98%, and, consistent, with our previous study [38] postinsertion of the TAT-lipopeptide into preformed liposomes had no significant impact on DXR release from liposomes (data not shown).

**Figure 2:**
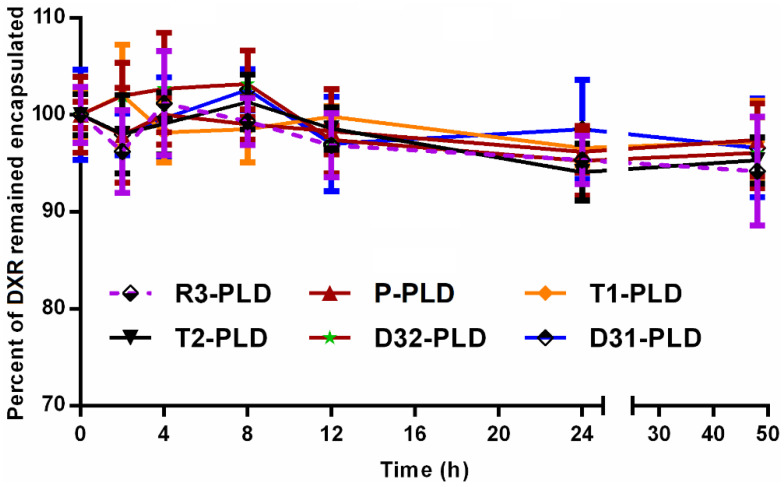
Leakage stability assessment of different liposomal preparations at 37 °C in the presence of fetal calf serum (FCS). Diluted samples of liposomes were transferred to a dialysis cassette and dialyzed against 100 mL of sucrose 10% supplemented with 30% FCS at 37 °C. At different time points, the amount of doxorubicin released to the dialysis medium was determined, and the amount of DXR remaining encapsulated was measured. Data represented as mean ± SD (*n* = 3).

All liposomes were stable during 48 h of incubation at 37 °C in the presence of FCS, and no significant difference in leakage stability between liposomal preparations was observed (Figure 2). In addition, all liposomal suspension showed no sign of colloidal instability during at least a month of storage at 4 °C under an argon atmosphere.

### 3.3. In Vitro Cellular Association of Peptide-Modified Liposomes

**Figure 3:**
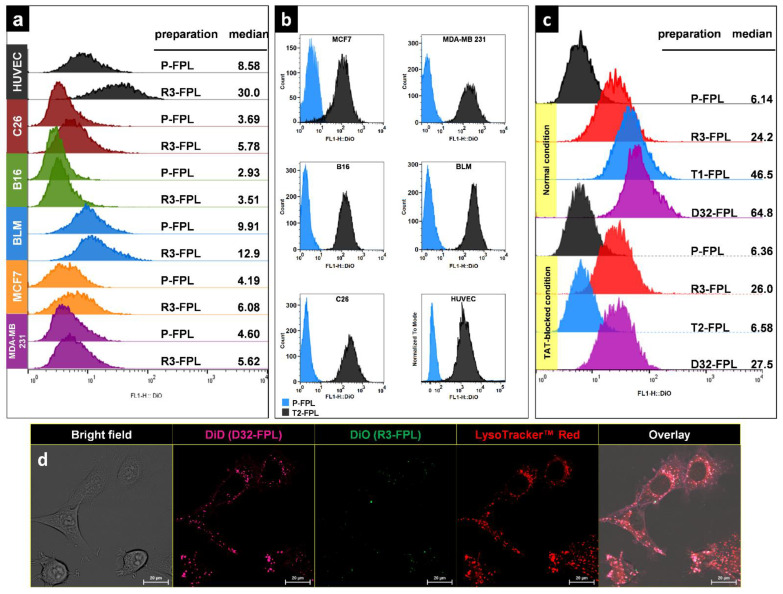
In vitro cellular association of peptide-modified liposomes. (**a**) represents the flow cytometry analysis of different cells exposed to either RGD-modified liposomes (R3-FPL) or plain liposomes (P-FPL). (**b**) represents the flow cytometry analysis of different cells exposed to either TAT-modified liposomes (T2-FPL) or P-FPL. (**c**) represents the cellular association of P-FPL, R3-FPL, T2-FPL, and dual-modified liposomes (D32-FPL) with HUVEC cells in normal and TAT-blocked conditions. (**d**) depicts the confocal live cell imaging of the cellular association of DiO-labeled R3-FPL and DiD-labeled D32-FPL concomitantly incubated with C26 cells, which were preincubated with LysoTracker Red to stain acidified organelles. In all studies, cells (105/ well) were exposed to 100 of nmol phospholipid/500 µL of each preparation, incubated for 3 h at 37 °C, washed, and analyzed. Scale bar: 20 μm.

The targeting ability of RGD-liposomes and TAT-liposomes against a variety of cancer cells, as well HUVEC cells, was assessed in vitro. Modification of liposomes with RGD enhances the association of liposomes only with HUVEC cells, whereas differences between the interaction of RGD-liposomes and plain liposomes with cultured cancer cells are negligible (Figure 3a). However, modification of liposomes with a TAT peptide increases the association of liposomes with both endothelial and tumor cells (Figure 3b).

In order to evaluate possible cross interaction between RGD and -igands on cell surface, HUVEC cells were exposed to different liposomal preparations under normal conditions and TAT-blocked conditions by heparin sulfate [48,49]. Figure 3c depicts that both RGD and TAT enhanced liposome-cell interaction under normal conditions, and once both ligands were decorated on FPLs, further designated as D32-FPLs, the dual-modified preparation interacted more with cells compared to their single-modified counterparts. The blocking of TAT resulted in a reduction in interaction to the level of RGD alone. These results reveal that the interaction of TAT and RGD on liposomes is independent, and the presence of both active ligands improves the cellular association of liposomes.

The functionality of D32-FPL against cancer cells was also visualized and compared with R3-FPL (RGD-targeted FPL) when both liposomes were incubated with a C26 cell line. Figure 3d clearly confirms the flow cytometry results where RGD-liposomes do not interact with C26 cells, while D32-FPL is internalized into cells via TAT-mediated endocytosis. Endocytosis was confirmed by co-localization of liposomal DiD with LysoTracker-stained red acidified organelles.

### 3.4. Intravital Microscopy

#### 3.4.1. RGD-Liposomes vs. TAT-Liposomes

**Figure 4:**
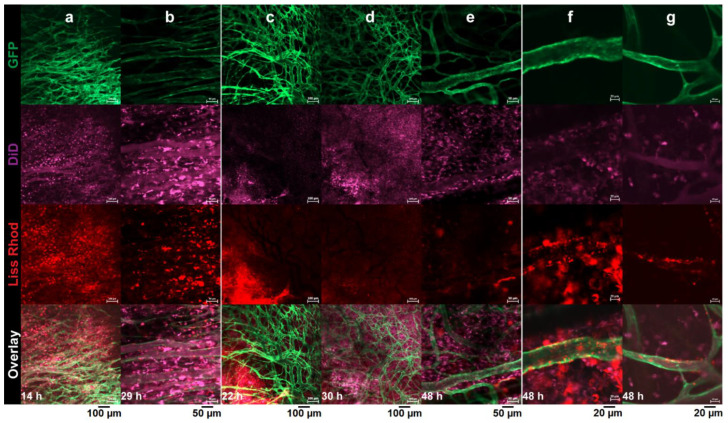
Intravital microscopy imaging of intratumoral behavior of Rho-PE-labeled (red) RGD-liposomes (R3-FPL) and DiD-labeled (purple) TAT-liposomes (T2-FPL) inside B16F0 tumors using the dorsal skin-fold chamber. While in some regions both liposomes exhibit almost identical cellular association (**a**,**b**), in some regions extravasated T2-FPL interacts more than extravasated R3-FPL with cells (**c**–**e**), while it is R3-FPL that can be seen associated with tumor vasculature (**f**,**g**). Mice were co-injected with 5 μmol of each liposomal preparation and images were captured at different time points after injection. Green fluorescence of endothelial cells due to eNOStag-GFP expression.

The intratumoral behaviors of RGD-liposomes and TAT-liposomes were evaluated and compared against each other by intravital microscopy imaging after an IV injection of a liposomal cocktail containing identical amounts of both liss-rhodamin-PE-labeled R3-FPL and DiD-labeled T2-FPL. Pictures captured from different parts of the tumor were evaluated with respect to cellular and vascular association of both injected peptide-modified liposomes. In some parts of the tumor (Figure 4a,b), both liposomes showed almost identical function. Both preparations showed identical extent of cellular association upon reaching the tumor interstitium, and both showed no sign of vascular targeting. In other regions, liposomes showed different behavior. While in Figure 4c–e, extravasated T2-FPLs are visible as punctate fluorescence spots, which indicates cellular association, R3-FPL exhibits no vascular targeting and limited cellular association and mainly displays a diffused distribution in spite of the presence of a high amount of extravasated red liposomes. However, in contrast to TAT-liposomes that show no association with tumor vasculatures (Figure 4a–g), R3-FPLs show some interaction with the tumor vasculature (Figure 4f–g). Lack of interaction of R3-FPL with tumor vessels before extravasation or with tumor cells after extravasation in some regions of tumor indicates a heterogeneous targeting ability of RGD-liposomes towards integrins, while TAT-liposomes display a certain and homogenous ability to interact with cells upon reaching the tumor interstitium, regardless of the location of extravasation. 

#### 3.4.2. Dual-Targeted Liposomes

**Figure 5:**
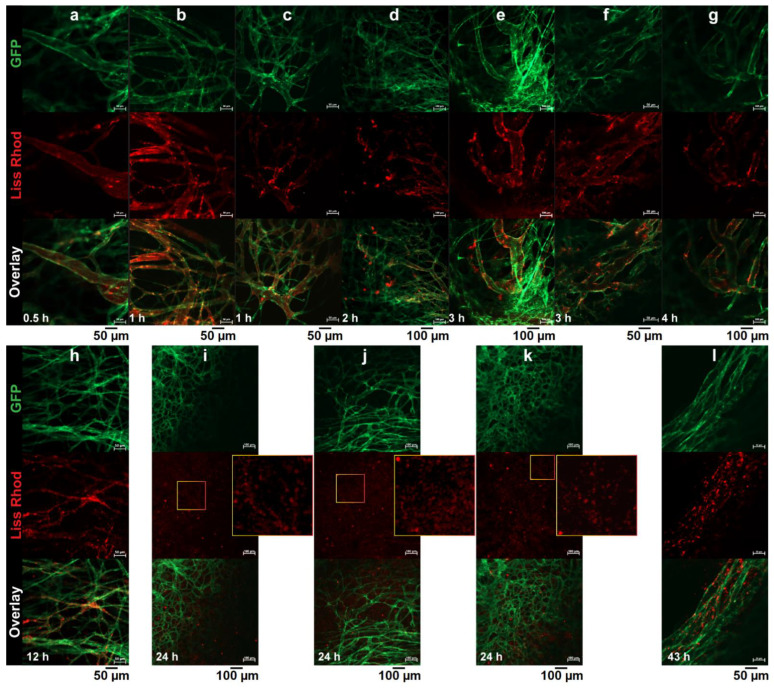
Intravital microscopy imaging of intratumoral behavior of Rho-PE-labeled (red) dually targeted liposomes (D32-FPL) in targeting tumor vasculature (**a**–**h**) or in extravascular association with cells (**i**–**l**) inside B16F0 tumors using the dorsal skin-fold chamber upon an IV injection of 5 μmol of liposomal lipids. Images were captured at different time points after injection. Green fluorescence of endothelial cells due to eNOStag-GFP expression.

Visualization of tumor endothelium targeting of dual-modified liposomes revealed a substantial interaction of these liposomes with the tumor vasculature, which started soon after injection (Figure 5a–h). Interestingly, the extent of vascular targeting of these liposomes was remarkably greater than what was observed with the RGD-liposomes (Figure 4). In addition to massive interaction with tumor vessels, TAT-modified liposomes were also found to be associated with other tumor-associated cells (Figure 5i–l), showing their capability of delivering drugs into cells residing inside the tumor interstitium.

#### 3.4.3. Dual-Targeted Liposomes vs. Single-Targeted Liposomes

**Figure 6:**
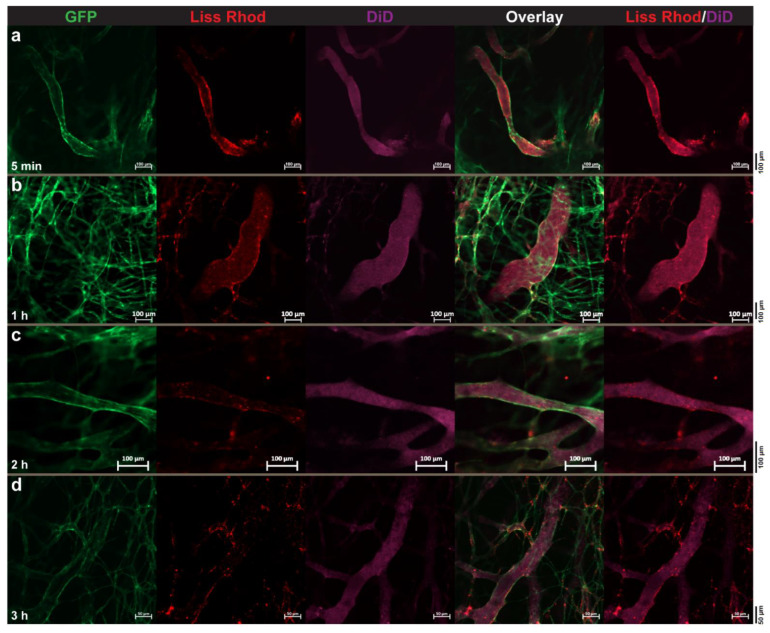
Intravital microscopy of intratumor behavior of Rho-PE-labeled (red) dually targeted-liposomes (D32-FPL) mixed and injected with DiD-labeled TAT-liposomes (T2-FPL). Images were captured at different time points of 5 min to 3 h (**a**–**d**) after injection show association of D32-FPL (red) with tumor vessels while no significant association between T2-FPL (purple) and tumor vasculature could be seen. Mice were injected with an IV injection of 5 μmol of each liposomal lipids. Green fluorescence of endothelial cells due to eNOStag-GFP expression.

In order to evaluate and compare the targeting ability of dual-targeted liposomes with liposomes modified with TAT or RGD peptides alone, intravital imaging of tumors after an IV injection of a liposomal cocktail containing the dually targeted liposomes and single-targeted preparations was performed. In Figure 6, we show high association of D32-FPLs with the tumor vasculature, while no pronounced vascular interaction was observed with T2-FPL. These results indicate the determining role of RGD in vascular targeting of D32-FPL, while the presence of TAT, as observed earlier (Figure 4), hardly led to tumor vascular interaction.

**Figure 7:**
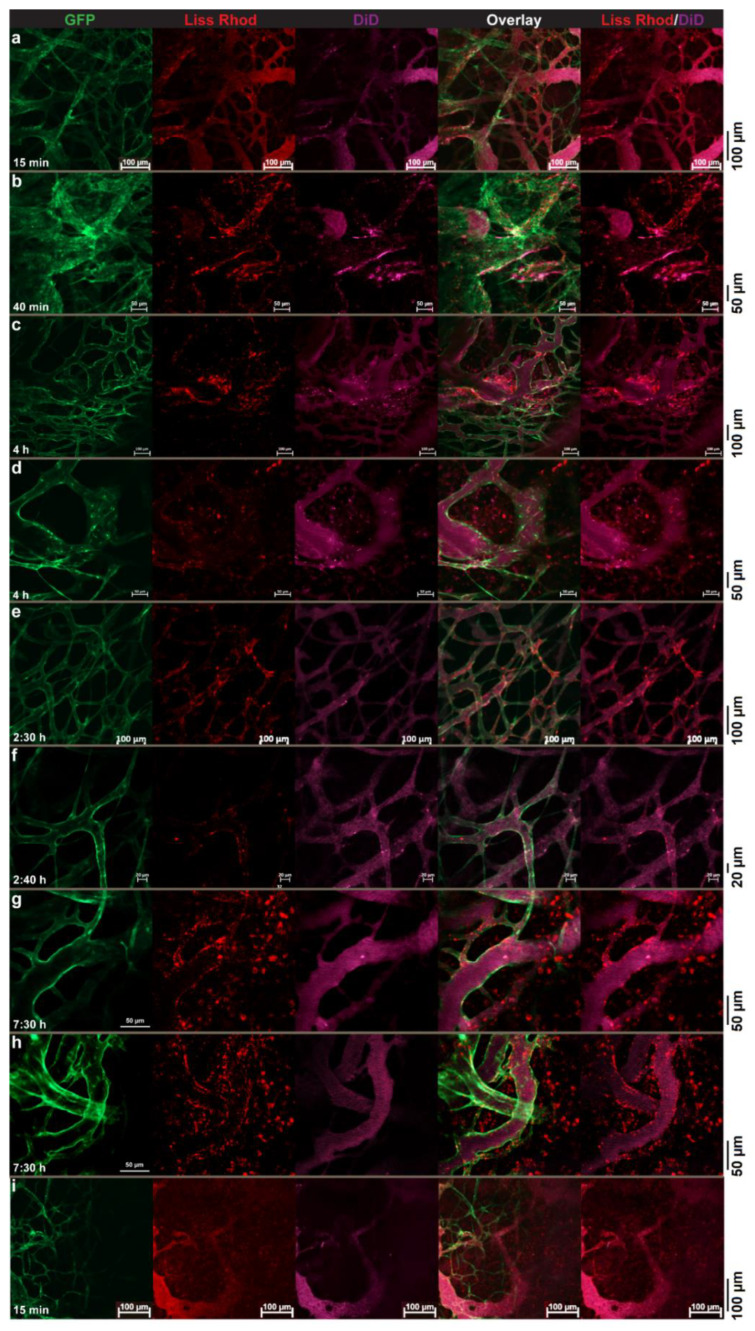
Intravital microscopy of intratumor behavior of Rho-PE-labeled (red) dually targeted liposomes (D32-FPL) mixed and injected with DiD-labeled RGD-liposomes (R3-FPL). While in some regions both liposomal preparations represent identical vascular targeting (**a**,**b**) in more captured images (**c**–**h**) D32-FPL exhibits superior vascular targeting compared to R3-FPL that in some regions failed to interacts with tumor vessels (**g**,**h**). Images (**g**–**i**) show cellular association of extravasated D32-FPL while extravasated R3-FPL does not interact with cells inside tumor interstitium. Mice were injected with an IV injection of 5 μmol of each liposomal lipids and images were captured at different time points after injection. Green fluorescence of endothelial cells due to eNOStag-GFP expression.

In another study, wherein mice received a mixture of D32-FPL and R3-FPL, the dual-modified preparation exhibited a superior targeting activity towards tumor structures. While in some regions (Figure 7a,b), both preparations exhibited almost identical interactions with tumor vasculature or tumor cells and were mostly seen co-localized to an almost identical extent, in most regions, D32-FPL dominates the vascular interactions, whereas minor (Figure 7c–f) or no (Figure 7g,h) vascular associations could be seen with R3-FPL. In addition to vascular targeting, D32-FPL also exhibited significant cellular association inside the tumor interstitium, where no cellular association of R3-FPL could be traced (Figure 7g–i). In Figure 7i, in which significant amounts of both liposomes could be seen leaking out from vessels, only D32-FPLs interacted with cells upon leaving the vessel, and R3-FPLs exhibited no affinity towards cells inside that part of tumor.

### 3.5. Biodistribution of PLDs in Tumor-Bearing Mice

**Figure 8:**
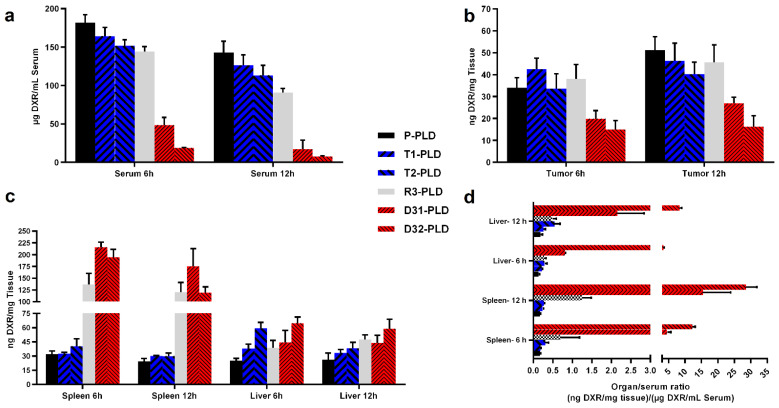
Biodistribution of PLDs at different time points after a single IV injection of 15 mg/kg of liposomal DXR in the serum (**a**), tumor (**b**), and spleen and liver (**c**) of C57 mice bearing B16 tumors. Panel (**d**) represents the affinity of different liposomes to spleen and liver at different time points. Data represent mean ± SD (*n* = 4).

The biodistribution of different PLDs in the C57 mouse model of the B16 tumor was evaluated in a dose of 15 mg/kg of liposomal DXR. Modification of liposomes with peptides resulted in a slightly lower DXR level in the serum of mice at 6 h or 12 h after injection (Figure 8a). While T1-PLD and P-PLD exhibited statistically identical blood levels at both studied time points (*p* > 0.05), T2-PLD or R3-PLD was 1.3 times lower compared to P-PLD at 6 h post injection (*p* < 0.05). At 12 h, injection with T2-PLD and R3-PLD resulted in 1.3 (*p* < 0.05) and 1.5 (*p* < 0.05) times lower serum levels of DXR compared to P-PLD, respectively. However, the slight reduction in blood levels of single-modified preparations did not result in a different degree in tumor accumulation, and all preparations accumulated in statistically identical amounts in the tumors (Figure 8b). These results are consistent with our previous observation in a BALB/c mouse model of C26 tumor [31,38], implying that modification of liposomes with the TAT or the RGD peptides used in our studies did not result in a dramatic enhanced clearance rate of liposomes. However, when both peptides were decorated on the surface of liposomes, the clearance rate of dual-modified liposomes increased dramatically. Compared to mice injected with P-PLD, mice receiving D31-PLD had 3.8 and 8 times lower DXR levels in serum at 6 and 12 h after injection, respectively. Injection with D32-PLD resulted in 9.7 and 19 times lower DXR levels in blood compared to injection with P-PLD at 6 and 12 h post injection, respectively. Comparison of blood levels of TAT-liposomes reveals that T1-PLD created 1.2 (*p* < 0.05) and 1.1 times (*p* > 0.05) higher blood levels than T2-PLD at 6 and 12 h after injection, respectively. This implies that doubling the number of TAT peptides on the surface of liposomes from 100 to 200 peptide/liposome does not have a remarkable impact on the pharmacokinetics of TAT-modified liposomes. However, when such an increase in the density of the TAT peptide was applied on RGD-liposomes, the clearance rate increased dramatically. The DXR level in the serum of mice receiving D32-PLD was only about 40% (*p* < 0.05) of what observed after injection with D31-PLD at both studied time points. This suggests a synergistic activity of RGD and TAT in enhancing the clearance rate of dual-modified liposomes.

In order to analyze the uptake of liposomes by liver and spleen, we presented the data in two ways. Figure 7c represents the absolute concentration of DXR in organs of mice that received different preparations. Figure 8d represents the ratio of DXR in organ-to-serum for qualitative comparison of liposome affinity towards spleen and liver. It should also be taken into account that both of these organs are highly vascularized and contain a significant amount of blood. Therefore, a part of the quantified drug in these organs comes from blood, unless the organs are thoroughly perfused before homogenization. Using the organ-to-serum ratio can magnify the differences in affinity of liposomes for these organs.

Through analysis of DXR levels in spleen and liver it, was found that the spleen plays the main role in the uptake and clearance of RGD-decorated liposomes from blood (Figure 8c,d). It was also found that while the affinity and uptake of T1-PLD and T2-PLD by spleen was identical with P-PLD, there is a direct correlation between the density of TAT on liposomes and liposome uptake by liver, suggesting a role for TAT in liver uptake of TAT-decorated liposomes. As can be seen in Figure 8c,d, elevated uptake of dual-modified liposomes indicates a remarkable affinity towards both spleen and liver.

Therefore, we suggest that that the observed involvement of both liver and spleen results from the presence of both ligands on the surface of dual-modified liposomes, which could explain the fast clearance rate of these preparations compared to non-targeted or single peptide-modified liposomes. As the result of fast clearance, D31-PLD and D32-PLD showed the lowest tumor accumulation (Figure 8b), which was only 0.5 and 0.3 of what observed with injecting P-PLD, respectively.

### 3.6. Therapeutic Efficacy

**Figure 9:**
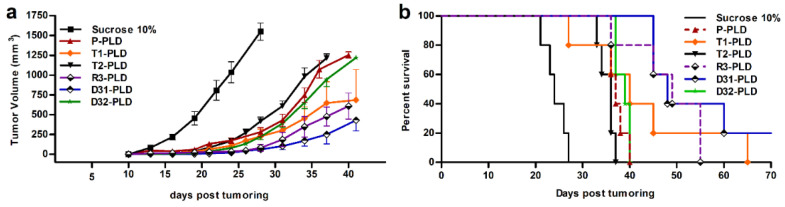
Therapeutic efficacy of various liposomal preparations: (**a**), tumor growth rate and (**b**), survival curve of female C57 mice bearing B16 tumors after an IV administration of 15 mg/kg of liposomal doxorubicin or sucrose 10% on day 10 after tumor inoculation. Data represented as mean ± SD (*n* = 5).

Therapeutic efficacy of different targeted and non-targeted PLDs was evaluated in a murine B16 melanoma tumor model. All PLDs were significantly more effective than 10% sucrose that had a median survival time of 24 days, and no animals survived to 26 days (Figure 9). Tumor growth curve analysis (Figure 9a) revealed that while P-PLD, T2-PLD, and D32-PLD were virtually identical with respect to an effect on tumor growth rate and resulted in medium survival times (MST) of 37, 36, and 39 days, respectively, T1-PLD, R3-PLD, and D31-PLD were more effective in slowing or halting B16 melanoma growth. Among all PLDs, only treatments with D31-PLD and R3-PLD significantly increased the survival time of treated mice compared to treatment with P-PLD (*p* < 0.05) (Figure 9b). Treatment with D31-PLD and R3-PLD resulted in identical MST, but mice receiving D31-PLD showed a slower tumor growth rate (*p* > 0.05). Besides, while no mice survived on day 55 in groups that were treated with R3-PLD, 20% of D31-PLD treated mice were cured and showed complete elimination of tumors.

## 4. Discussion

Dual functionalization of liposomes with two ligands in order to improve tumor targeting is being investigated. So far, dual-ligand-modified liposomes have been designed to improve the selectivity and cooperative uptake of nanoparticles by specific tumor cells [50,51,52,53,54] or tumor vasculatures [55,56,57] or to overcome biological barriers, particularly to enhance transport across the blood–brain barrier (BBB) and cellular delivery into brain malignancies [58,59,60,61,62,63]. The importance and benefits of these strategies have been well reviewed by Zhu et al. [64] and Belfiore et al. [65]. Except for the targeting of brain malignancies, in which each ligand has an independent function against different cells, where one ligand such as transferrin is responsible for translocation across the BBB and the other paired ligand-like p-aminophenyl-α-D-mannopyranoside [58], anti-amyloid beta peptide antibody [59], or CPP-like R9F2 [60], TAT [61], penetratin [62], or octaarginine [63] are responsible for the cellular entry of payload into malignant cells. Other studies have mostly been aimed at enhancing drug delivery into one kind of cell, where two ligands are either independently increasing the chance of recognition of certain cells that exhibit variable degrees of receptor expression or are dependently assisting enhanced interaction and uptake of targeted-nanoparticles by the targeted cells. For example, Mei and coworkers [50] designed a multistage dual modified liposome with RGD and TAT to enhance targeting tumor cells after passive accumulation inside the tumor. In their liposomes, the RGD peptide was conjugated to DSPE-PEG 3500 (DSPE-PEG3500-RGD), and the TAT peptide was conjugated to DSPE-PEG 1000 (DSPE-PEG1000-TAT). Both ligands were shielded by the addition of 8 mol% of a longer cleavable DSPE-PEG 5000, which cleaves in the presence a reducing agent such as L-cysteine. While long PEG chains protect ligands from being exposed during circulation that elongates circulation of liposomes in blood, cleavage of PEG 5000 upon administration of L-cysteine when liposomes are accumulated in the tumor leaves ligands exposed to interacting with tumor cells. In another study, Tang et al. [53] co-modified liposomes by transferrin and TAT and found a synergistic enhanced cellular uptake of dual-modified liposomes compared to single-modified preparations when tested on HepG2 cells that expressed a substantial transferrin receptor. However, when the expression of the transferrin receptor is low, such as in HUVEC, dual-modified liposomes and TAT-modified liposomes interact to the same extent with HUVEC cells. These studies showed that presence of two ligands increases the probability of liposomes interacting with cells inside tumor.

In some studies, it was claimed that dual-ligand modification could increase targeting selectivity or specificity; Saul et al. [51] modified liposomes with folic acid and a monoclonal antibody against an epidermal growth factor receptor (EGFR) for targeting tumor cells in a selective manner to reduce off-targeting. They claimed that their DXR-loaded dual-modified liposomes can only be cytotoxic when both folate and EGFR receptors are present while sparing off-target cells expressing no or only one of the targeted receptors. Another example is liposomes modified with glycyrrhetinic acid (GA) and peanut agglutinin (PNA) that improves the selective drug delivery to malignant liver tumors [54].

Similar to the dual-modification of liposomes against tumor cells, some studies focused on increasing the specificity of the targeted liposomes against tumor vasculature by exploiting two vascular-specific ligands. It has been found that co-modification of liposomes decorated with Ala-Pro-Arg-Pro-Gly (APRPG containing PRP motif for targeting vascular endothelial growth factor receptor-1 (VEGFR-1)) with either Gly-Asn-Gly-Arg-Gly (GNGRG containing NGR motif for targeting CD13 receptor) [56] or Gly-Arg-Gly-Asp-Ser (GRGDS containing RGD motif) [57] augments liposome affinity towards tumor vasculature [56,57]. In another study, Kluza and coworkers [55] showed that modification of liposomes with the two vascular targeting ligands galectin-1-specific anginex (Anx) and RGD results in enhanced specificity against tumor endothelium [55]. Ex vivo image analysis revealed that about 53 ± 6% of dual modified liposomes that were seen in the tumor were co-localized with the tumor endothelium of the B16F10 tumor model, which was significantly higher than 43 ± 9% (*p* = 0.043) and 28 ± 8% (*p* = 0.0001) of what was calculated when liposomes modified with only Anx or RGD peptides were injected, respectively. However, RGD-liposomes that exhibited a threefold slower clearance rate than the dual-modified liposomes accumulated more in tumors.

**Figure 10:**
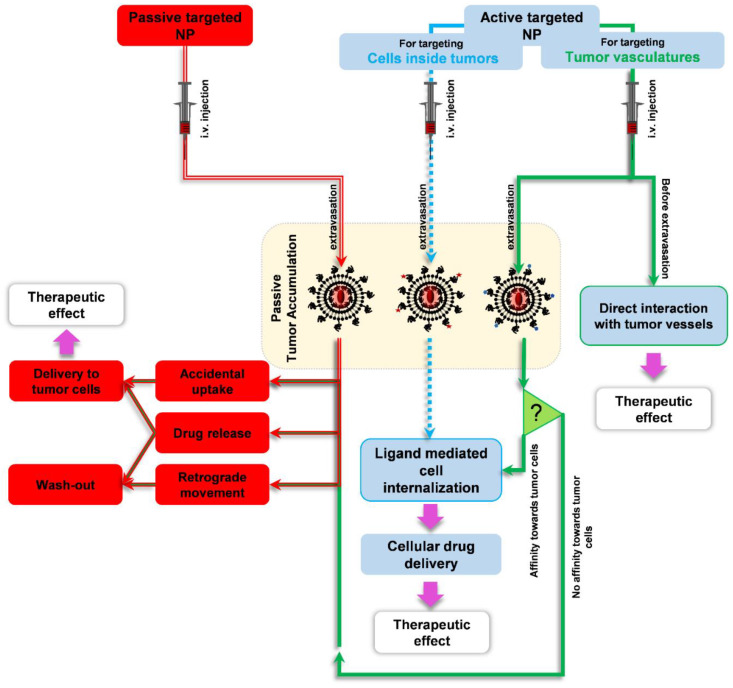
Schematic of targeting tumor tissue by means of passive or active targeted nano-particles (NP). A passive-targeted NP (red double line) requires passive extravasation and accumulation inside the tumor where the NP can release the payload, accidently being taken up by cells or being washed out from the tumor. An active-targeted NP that is designed to interact with cells inside the tumor (blue square dashed dot line) still requires EPR-based tumor accumulation similar to a passive-targeted NP. However, in contrast to a passive-targeted NP, a targeted NP can actively interact with cells expressing the target marker and consequently enter the targeted cell. When a vascular-targeted NP (green solid line) passes through a tumor, two scenarios may happen; this NP might recognize vascular endothelial cells and subsequently be taken up, or it may still passively extravasate into the tumor interstitium. If the targeted vascular marker is also expressed in tumor cells, then vascular-targeted NPs can actively interact with and be internalized by tumor cells. When tumor cells do not express the vascular marker, vascular-targeted NPs have the same fate as plain and non-targeted NPs have.

It has to be taken into account that in experimental tumor models, the majority of tumor accumulation of a nano-sized particle, either a ligand-modified active targeted one or a passive targeted plain one, is achieved through passive extravasation into the tumor interstitium. In fact, upon injection of a vascular-targeted liposome, a significant fraction of injected preparation, which is not little, will extravasate and accumulate inside the tumor interstitium due to such passive accumulation. This has been shown in Figure 4c–e, where a significant amount of RGD-modified liposomes could be seen inside the tumor interstitium without being associated with cells. Therefore, in extravascular regions of a tumor, a vascular-targeted nanoparticle behaves as a non-targeted particle unless the targeted vascular marker is also expressed on tumor cells or liposomes are equipped with a complementary function to interact with cells inside the tumor interstitium. This is schematically illustrated in Figure 10, where the fates of plain and ligand-modified nanoparticles in targeting tumors are compared.

Based on this, it was hypothesized that the dual modification of liposomal doxorubicin with two independent functionalities, one for the intravascular targeting of tumor vasculature via the RGD peptide and the other for extravascular targeting and internalizing liposomes into cells inside the tumor via a TAT peptide motif, could increase the targeted area of a tumor and hence reduce the negative impact of tumor heterogeneity compared to targeting the tumor with liposomes modified with single peptide. Therefore, the primary goal of this study was to evaluate and achieve the proof-of-principle for this hypothesis. It is worth mentioning that, in contrast to those dual-modified liposomes that essentially require both targeted receptors to be present on the surface of target cells for being uptaken and to achieve a selective targeting [51,54] or to benefit from a synergistic effect [50,52,53,55,56,57], the independent capability of each ligand to interact and mediate the uptake of liposomes by cells expressing one or both receptors is crucial. It has been confirmed in our in vitro studies that cellular association of RGD and TAT are independent from each other in our experimental setting. First, the tumor-targeting behavior of single-targeted liposomes was evaluated and visualized in vivo by intravital microscopy. We observed that vascular-targeted RGD-liposomes (R3-FPL) exhibit a spatially heterogeneous targeting ability against both tumor vasculature and cells inside the tumor interstitium, implying a heterogeneous expression of α_v_β_3_ integrin on tumor cells and tumor vasculature, which is consistent with what Seguin et al. [30] and Boles et al. [66] have observed. Kluza and coworkers [55] have also observed a high degree of variation in the targeting of tumor vasculature when a group of mice bearing the B16F10 tumor received RGD-liposomes, whereas those injected by Anx-liposomes or dual modified ANX/RGD-liposomes exhibited more inter-group homogeneity with respect to association of liposomes with tumor vasculature. Considering the co-administered TAT-liposomes (T2-FPL) as a positive control of cellular association, several regions of the tumor interstitium were found negative with respect to α_v_β_3_ expression, in which only T2-FPL succeeded in interacting with cells, while R3-FPL failed to interact with cells after extravasation. This is a strong indication that a significant part of extravasated RGD-liposomes behave like plain liposomes upon reaching the tumor interstitium.

Regarding the intratumor behavior of TAT-liposomes, two significant observations were made. First, TAT-liposomes exhibited a more homogenous and predictive function upon reaching the tumor interstitium and were found to be associated with cells inside tumor, similar to what was observed in vitro. The other significant observation was the lack of interaction between T2-FPL and tumor vessels in spite of in vitro results that show that TAT enhances liposome association with HUVEC cells (Figure 3b,c) or what observed by others [53]. This is another bit of evidence supporting our previous statement that the TAT peptide sequence used in the current study behaves differently in static or dynamic conditions once installed on the surface of PEGylated liposomes. It seems that TAT residues are quiescent under dynamic conditions, such as in the circulation, but become active under static conditions, such as in the tumor interstitium or in a plate in vitro. Pharmacokinetics of TAT-modified liposomes also support this hypothesis, where despite the positive charge of the TAT peptide, no enhanced clearance rate and uptake by spleen and liver representing RES was seen. In fact, this was the reason for choosing the TAT sequence as a complementary ligand next to the RGD peptide. In brief, this part of our study reveals that RGD can direct liposomes to the tumor vasculature and, to some heterogeneous extent, cells inside tumors, but TAT can only increase cellular uptake of liposomes after extravasation but in a predictive manner.

The next step was to evaluate the intratumoral behavior of dual-modified liposomes and compare it with co-administered single peptide-modified liposomes. Intravital images showed high and substantial affinity of D32-FPL towards tumor vasculatures, and liposomes were also found to be actively associated with cells inside the tumor upon extravasation. It was found that the affinity of D32-FPL for the tumor vasculature was regulated by the RGD ligand since the co-administered T2-FPL again showed no vascular association. Co-administration of D32-FPL and R3-FPL revealed two significant advantages of D32-FPL over R3-FPL. First, there was the enhanced vascular targeting of D32-FPL compared to R3-FPL. While TAT showed no function against tumor endothelium, the addition of TAT to R3-FPL significantly enhanced vascular targeting of D32-FPL and, interestingly, superseded R3-FPL with respect to interaction with exposed α_v_β_3_ on tumor vasculature. Given the active function of TAT residue on the surface of PEGylated liposomes under static conditions and their inactivity under dynamic conditions, it is likely that that a primary weak interaction and stabilization of liposomes on a cell surface was being consolidated by the interaction of TAT residues with heparan sulfate proteoglycans that were expressed ubiquitously on the cell surface, which consequently resulted in stronger cellular association. Results reported by Kibria and coworkers [52] can support this hypothesis. They decorated their dual modified liposomes with 5 mol% DSPE-PEG 2000-RGD and 2 mol% stearylated octaarginine, which is a CPP. In such liposomes, while RGD is fully exposed on surface of the liposome, PEG chains surround the octaarginine, which is directly attached to the lipid bilayer. It was found that while cellular uptake or transfection efficacy of single-ligand-modified preparations were negligible, dual-ligand-modified liposomes showed an enhanced uptake and transfection efficacy in α_v_β_3_-expressing cells. It is likely that, similar to the in vivo behavior of D32-FPL, the primary interaction of RGD moiety allows the hidden octaarginine moieties to step in and accelerate the cellular internalization of liposomes.

The second important observation is the capability of D32-FPL to associate with cells after extravasation. In images showing extravasation of both R3-FPL and D32-FPL, mostly D32-FPL interacted with cells, while the interaction of R3-FPL with cells was hardly observed. These observations provide experimental proof of the hypothesis that dual-modified liposomes are capable of targeting tumor vasculature via the RGD ligand while circulating in blood, and upon reaching the tumor interstitium, the TAT motif enhances cellular interaction with cells residing there. In other words, compared to single-ligand-modified liposomes, dual-modified liposomes are able to cover more regions of a tumor for cellular drug delivery, and drug delivery to a tumor is more efficient.

To see if such advantages of dual modifications compared to single-peptide modification can be translated into better therapeutic outcomes, liposomes were further studied in vivo with respect to biodistribution and therapeutic efficacy in a mouse model of a B16 tumor.

The biodistribution study reveals that, while R3-FPL and liposomes with different densities of TAT (T1-FPL and T2-FPL) circulate long in blood, and only a minor reduction in circulation time was observed, the presence of both peptides synergistically increases the clearance rate of dual-modified preparations (D32-FPL and D31-FPL). Such fast clearance rates could also be seen in intravital images, where the intravascular intensity of labeled D32-FPL dropped rapidly and much faster than their co-administered single-peptide-modified counterparts, which prompted us to include D31-PLD in other in vivo studies. It is likely that, comparable to interaction in tumor regions, where D32-FPL exhibits greater avidity than R3-FPL for integrin-expressing endothelial cells, the direct interaction of dual-modified liposomes with integrin receptors expressed in the liver and spleen is enhanced. If so, this explains the increased uptake in the D32-FPL preparations from blood compared to other preparations. In addition to this, as indicated earlier and reported previously [31,38], in the absence of RGD, the peptide doubling of TAT density does not change the pharmacokinetics of TAT-liposomes significantly. However, in the presence of the RGD peptide, the doubling of TAT density (i.e., from 100 to 200 peptides/liposome on dual-modified liposomes) significantly enhanced the clearance rate of liposomes by the RES. In our previous study in BALB/c mice, liposomes with different densities of TAT revealed identical accumulation in both liver and spleen at 24 and 48 h after injection. However, in the current study we report that at earlier time points of 6 or 12 h after injection, TAT modification slightly increases the liver uptake directly proportionally with the peptide density on liposomes, while no sign of spleen function in the clearance of TAT-PLD was observed. Given the dominant role of the spleen in the uptake of R3-PLD, it could be concluded that presence of both ligands on the surface of liposomes involves both spleen and liver in recognition and the clearance of dual-modified preparations, thereby accelerating clearance rate. Given the role of protein corona associated with nanoparticles in governing the fate and performance of nanoparticles in a biological environment, proteomics analysis of protein corona associated with these liposomes is in process to find an explanation about the role of blood-derived proteins on accelerating the clearance rate of dual-modified liposomes.

The fast clearance rate of dual-modified liposomes resulted in lower delivery of DXR to tumors. However, while DXR delivery to tumor by D31-PLD after 12 h of injection was half of what was delivered by non-targeted or single-peptide-modified preparations, the antitumor efficacy was significantly greater than P-PLD, T2-PLD, and D32-PLD. Additionally, in treatments with R3-PLD, T1-PLD, or D31-PLD, although the differences in animal survival were not statistically significant, a better trend in slowing tumor growth rate was observed in mice treated with D31-PLD.

## 5. Conclusions

This study reveals that while TAT-modified liposomes exhibit predictable tumor targeting, although mainly to cells inside the tumor interstitium, RGD-modified liposomes are able to target both tumor vasculature and cells inside the tumor, but heterogeneously and therefore in a less predictable nature. The addition of TAT-peptides to RGD-modified liposomes not only resulted in the homogenous cellular association of liposomes inside the tumor but, unexpectedly, also improved vascular targeting and together increased the targeted area. As a consequence, these peptide-modified liposomes containing doxorubicin show enhanced therapeutic efficacy against a B16 tumor model. However, both of the studied dual-modified preparations exhibit poor pharmacokinetics, making these formulations less reliable candidates for the delivery of cytotoxic agents to solid tumors. Although we have not seen a significant indication of side effects when monitoring animal weights after injection (data not shown), the accumulation of DXR in organs such as the liver and spleen may lead to detrimental side effects. However, given the enhanced vascular interaction, this combination could be a great candidate for vascular delivery of nontoxic payloads or contrast agents for therapeutic or diagnostic purposes. In summary, dual modification of liposomes with a vascular targeting ligand and a TAT peptide was found beneficial. However, more studies on optimizing ligands densities of RGD/TAT or using other vascular-specific ligands that do not provoke RES uptake may improve or result in more favorable pharmacokinetics for targeting a tumor.

## Figures and Tables

**Table 1:** Description and colloidal properties of PLDs.

Preparation Name	Density of Surface-Inserted Peptides (Peptides/ Liposome)		ζ—Average (nm)	PDI ^1^	ζ Potential (mv)
	RGD	TAT			
Plain-PLD	0	0	105 ± 4.3	0.085 ± 0.01	−27.1 ± 2.1
T1-PLD	0	100	102 ± 5.2	0.073 ± 0.01	−25.3 ± 1.7
T2-PLD	0	200	106 ± 6.3	0.091 ± 0.01	−24.2 ± 1.4
R3-PLD	300	0	101 ± 2.4	0.048 ± 0.01	−26.3 ± 1.8
D31-PLD	300	100	100 ± 3.8	0.052 ± 0.01	−24.9 ± 1.3
D32-PLD	300	200	104 ± 5.2	0.055 ± 0.02	−22.8 ± 0.8

^1^ Polydispersity index. Data represented as mean ± SD of three (*n* = 3) different measurements carried out for each sample in sucrose 10%.

## Data Availability

The data presented in this study are available in the article.

## Abbreviations

RGD: Arginine–Glycine–Aspartic acid, TAT: transactivator of transcription, PEG: Polyethylene glycol, DXR: doxorubicin, PLD: PEGylated liposomal doxorubicin, FPL: fluorescently labeled PEGylated liposomes, CPP: cell-penetrating peptide, P-PLD: Plain PEGylated liposomal doxorubicin, T1-PLD: PLD decorated with 100 TAT peptide on the outer surface, T2-PLD: PLD decorated with 200 TAT peptide on the outer surface, R3-PLD: PLD decorated with 300 RGD peptide on the outer surface, D31-PLD: PLD decorated with 300 RGD peptide and 100 TAT peptide on the outer surface, D32-PLD: PLD decorated with 300 RGD peptide and 200 TAT peptide on the outer surface, EPR: Enhanced Permeation and Retention.
